# Probiotics in irritable bowel syndrome: strain-specific effects, diet, and biomarker timing

**DOI:** 10.3389/fcimb.2026.1794927

**Published:** 2026-05-13

**Authors:** Zoila Mora Guzmán, Irma Leticia Bazán Salinas, Sonia Moreno-Cabral, Eduardo Pérez-Campos, María Teresa Hernández-Huerta, Hector Alejandro Cabrera-Fuentes

**Affiliations:** 1 Centro de Investigación Facultad de Medicina UNAM-UABJO, Facultad de Medicina y Cirugía, Universidad Autónoma “Benito Juárez” de Oaxaca, Oaxaca, Mexico; 2 División de Estudios de Posgrado e Investigación, Tecnológico Nacional de México/Instituto Tecnológico de Tijuana, Tijuana, Mexico; 3 División de Estudios de Posgrado e Investigación, Tecnológico Nacional de Mexico, Instituto Tecnológico de Oaxaca, Oaxaca de Juárez, Oaxaca, Mexico; 4 Secretaría de Ciencia, Humanidades, Tecnología e Innovación (SECIHTI), Faculty of Medicine and Surgery, Autonomous University “Benito Juárez” of Oaxaca, Oaxaca, Mexico; 5 Hospital General de Zona No.1 Dr. Demetrio Mayoral Pardo, Instituto Mexicano del Seguro Social (IMSS), Oaxaca, Oaxaca, Mexico; 6 R&D Group, Vice Presidency for Scientific Research and Innovation, Imam Abdulrahman Bin Faisal University, Dammam, Saudi Arabia

**Keywords:** diet–microbiota interactions, irritable bowel syndrome, probiotic metabolites, short-chain fatty acids, strain specificity

## Introduction

Irritable bowel syndrome (IBS) is a common disorder of gut–brain interaction characterized by abdominal pain, altered bowel habits, and substantial reduction in quality of life (Li X. et al., 2025). IBS has a multifactorial pathophysiology, involving alterations in gut microbiota composition and metabolism, epithelial barrier dysfunction, immune activation, visceral hypersensitivity, and dietary triggers (Cheung and Kenway, 2026). These interacting axes have positioned probiotics as a therapeutic intervention with strong biological plausibility, particularly through modulation of short-chain fatty acid (SCFA) production and mucosal signaling (Kezer et al., 2025; Liu et al., 2025).

Recent randomized controlled trials, including a study of SCFA-producing probiotic metabolites, have reported improvements in global symptom burden, and selected markers of intestinal barrier function in IBS. Some of these studies also documented changes in microbiota-related metabolites such as acetate, propionate, and butyrate, supporting the biological plausibility of microbiome-targeted strategies (McFarland et al., 2021; Li E. et al., 2025; Almalki et al., 2026; Maslennikov et al., 2026). However, these findings should be interpreted within the broader context of strain-resolved meta-analyses and clinical guidelines, which consistently indicate that probiotic efficacy in IBS is heterogeneous, strain-specific, and highly context dependent (Gordon et al., 2003; McFarland et al., 2021; Maslennikov et al., 2026). This heterogeneity is further compounded by variation in IBS subtype representation and diagnostic criteria across studies, as differences between IBS-D (diarrhea-predominant IBS), IBS-C (constipation-predominant IBS), IBS-M (mixed IBS), and IBS-U (unsubtyped IBS), as well as between Rome III and Rome IV definitions, may influence both pathophysiological interpretation and treatment response (Gordon et al., 2003; Lacy et al., 2021; Vasant et al., 2021; Almalki et al., 2026). While these findings contribute to the expanding literature on probiotics in IBS, their interpretation and translation into routine clinical practice warrant a more nuanced and critical appraisal. Accumulating evidence from clinical guidelines and strain-resolved meta-analyses indicates that probiotic efficacy in IBS is neither uniform nor class-wide, but instead depends critically on strain specificity, dietary context, and methodological rigor in biomarker assessment (McFarland et al., 2021; Goodoory et al., 2023; Almalki et al., 2026). Current evidence suggests that probiotic efficacy in IBS is better interpreted at the strain or formulation level than at the class level (**Table 1**).

**Table 1:** Strain-specific and formulation-specific evidence of probiotic efficacy in irritable bowel syndrome.

Strain/formulation	*Lactobacillus plantarum* 299v	*Bacillus coagulans* MTCC 5260	*Saccharomyces boulardii* CNCM I-745	Defined multistrain probiotic formulations	Non–strain-resolved probiotics
Representative supporting studies	Strain-resolved systematic reviews and network meta-analyses; representative RCT-based syntheses	Network meta-analysis and strain-focused evidence syntheses	Outcome-specific reviews and meta-analytic syntheses	Strain- and outcome-specific systematic reviews and meta-analyses	Clinical guidelines and broader meta-analytic evidence
IBS subtype(s) studied	Mixed IBS populations; subtype-specific reporting inconsistent across studies	Mostly mixed or unspecified IBS populations	Mixed IBS populations: subtype-specific evidence limited	Often mixed or unspecified IBS populations	Broad IBS populations
Diagnostic criteria reported	Rome III/IV variably reported across included trials	Diagnostic definitions variably reported	Rome criteria not consistently distinguished across studies	Rome III and Rome IV both represented in the literature	Mixed diagnostic approaches across trials
Dose/exposure reporting	Dose reporting generally available, but not always standardized across studies	Dose ranges differ across trials; per-strain exposure reporting not always harmonized	Dosing generally described, but cross-trial standardization is limited	Total CFU frequently reported, but per-strain viability and delivered dose often unclear	Often incompletely reported at the strain level
Typical intervention duration (weeks)	4-12	4-12	4-8	4-12	Variable
Primary IBS outcomes reported	Reduction in abdominal pain; improvement in global IBS symptoms	Improved responder rates for abdominal pain and overall symptom relief	Modest improvement in abdominal pain and selected global symptom outcomes	Variable effects on IBS Symptom Severity Score, bloating, abdominal pain, and global symptom relief	Inconsistent or non-reproducible symptom benefit
Overall evidence appraisal	Among the more consistently supported strain-level signals in IBS, although effect size remains modest	Promising strain-specific signal, but evidence remains context dependent	Supportive but less consistent than leading bacterial strains; may be more adjunctive than definitive	Evidence should not be generalized across products, as efficacy appears to be formulation-specific	Current evidence does not support broad class-wide inference
Key limitations	Endpoint heterogeneity, inconsistent subtype stratification, variable trial quality, and limited comparability across formulations	Considerable variation in dose, duration, symptom endpoints, and patient phenotype across studies	Benefits appear heterogeneous, and evidence across IBS subtypes remains insufficiently resolved	Composition-specific effects, incomplete strain-level reporting, inconsistent viability disclosure, and high between-study heterogeneity	Lack of full strain identifiers, insufficient reproducibility, and limited translational utility for guideline development
Quantitative evidence	RCT sample sizes typically n≈50–200; small-to-moderate effect sizes for global symptom relief and abdominal pain across meta-analyses	RCTs typically n≈40–150; moderate improvements in responder rates reported, but effect magnitude varies across studies	RCTs typically n≈50–150; small effect sizes with variability across endpoints	Highly heterogeneous RCTs (n≈50–300); effect sizes inconsistent and formulation-dependent	Variable sample sizes; no consistent or reproducible effect estimates across studies
References	(McFarland et al., 2021; Zhang et al., 2022; Goodoory et al., 2023; Xie et al., 2023)	(Zhang et al., 2022; Xie et al., 2023; AbdelQadir et al., 2024)	(Gu et al., 2022; Xie et al., 2023; Maslennikov et al., 2026)	(McFarland et al., 2021; Zhang et al., 2022; Xie et al., 2023; Almalki et al., 2026)	(Lacy et al., 2021; Vasant et al., 2021; McFarland et al., 2023; Almalki et al., 2026)

This table summarizes representative strain-specific and formulation-specific probiotic evidence in IBS based on systematic reviews, network meta-analyses, and guideline-informed literature appraisal. Reported effects should not be generalized across genera, species, or commercial products. Interpretation remains constrained by heterogeneity in IBS subtype classification, diagnostic criteria, dosing transparency, formulation composition, and outcome definitions. IBS, irritable bowel syndrome; IBS-D, diarrhea-predominant IBS; IBS-C, constipation-predominant IBS; IBS-M, mixed IBS; IBS-U, unsubtyped IBS; RCT, randomized controlled trial; CFU, colony-forming units. References listed in parentheses correspond to supporting literature, not strain identifiers. Quantitative summaries are approximate and derived from representative RCTs and meta-analyses; they are intended to contextualize magnitude rather than provide pooled estimates.

Probiotic studies in IBS increasingly combine patient-reported outcomes with biological endpoints, including SCFA-related measures, intestinal permeability indices, and inflammatory markers, thereby strengthening mechanistic credibility beyond symptom-based efficacy alone (McFarland et al., 2021; Goodoory et al., 2023; Li E. et al., 2025; Tanaka and Vécsei, 2025; Maslennikov et al., 2026). Nevertheless, the use of metabolite-focused formulations without detailed descriptions of the producing strains and their specific strain-level characteristics, along with incomplete reports on the identity and dosage of each strain, limits comparability and clinical translatability between studies. In addition, short post-intervention follow-up constrains inference regarding durability of response, and the absence of dietary stratification or control leaves open the possibility that background nutritional patterns contributed to both SCFA dynamics and symptom modulation. Collectively, these considerations highlight the need for next-generation trials that integrate strain specificity, dietary context, and longitudinal biomarker timing to translate mechanistic signals into precision-guided clinical practice.

Despite mechanistic promise, translation of SCFA-linked probiotic interventions to clinical IBS management is constrained by three recurring design limitations: inadequate temporal biomarker resolution, uncontrolled dietary substrate exposure, and insufficient transparency into strain- and dose-level interventions. Beyond its classification as a disorder of gut–brain interaction, IBS represents a clinically tractable human model of disrupted host–microbial metabolic signaling, characterized by altered SCFA availability, epithelial energy metabolism, and low-grade immune activation. These pathways intersect closely with mechanisms central to metabolic disease pathogenesis, including compromised epithelial barrier function, persistent low-grade inflammation, and disrupted microbial metabolite–host signaling. From this perspective, IBS provides a valuable framework for interrogating how probiotic-derived metabolites modulate host metabolic physiology in humans.

In this Opinion, we argue that the insights gained from SCFA-focused probiotic interventions in IBS are directly relevant to the broader field of metabolic disease research. At the same time, we are exposing critical methodological limitations that must be addressed to translate probiotic metabolite science into reproducible, precision-guided clinical strategies.

The evaluative guidelines that inform this perspective, strain specificity, dietary context, biomarker timing, dose transparency, and harmonization of results, are derived from established clinical guidelines, strain meta-analyses, and methodological recommendations in probiotic research. Therefore, rather than establishing a formalized scoring framework, we present a structured expert synthesis to highlight the recurring methodological determinants that shape translational reproducibility in probiotic trials for IBS.

## A translational framework for probiotic trial design in IBS

**Figure 1:**
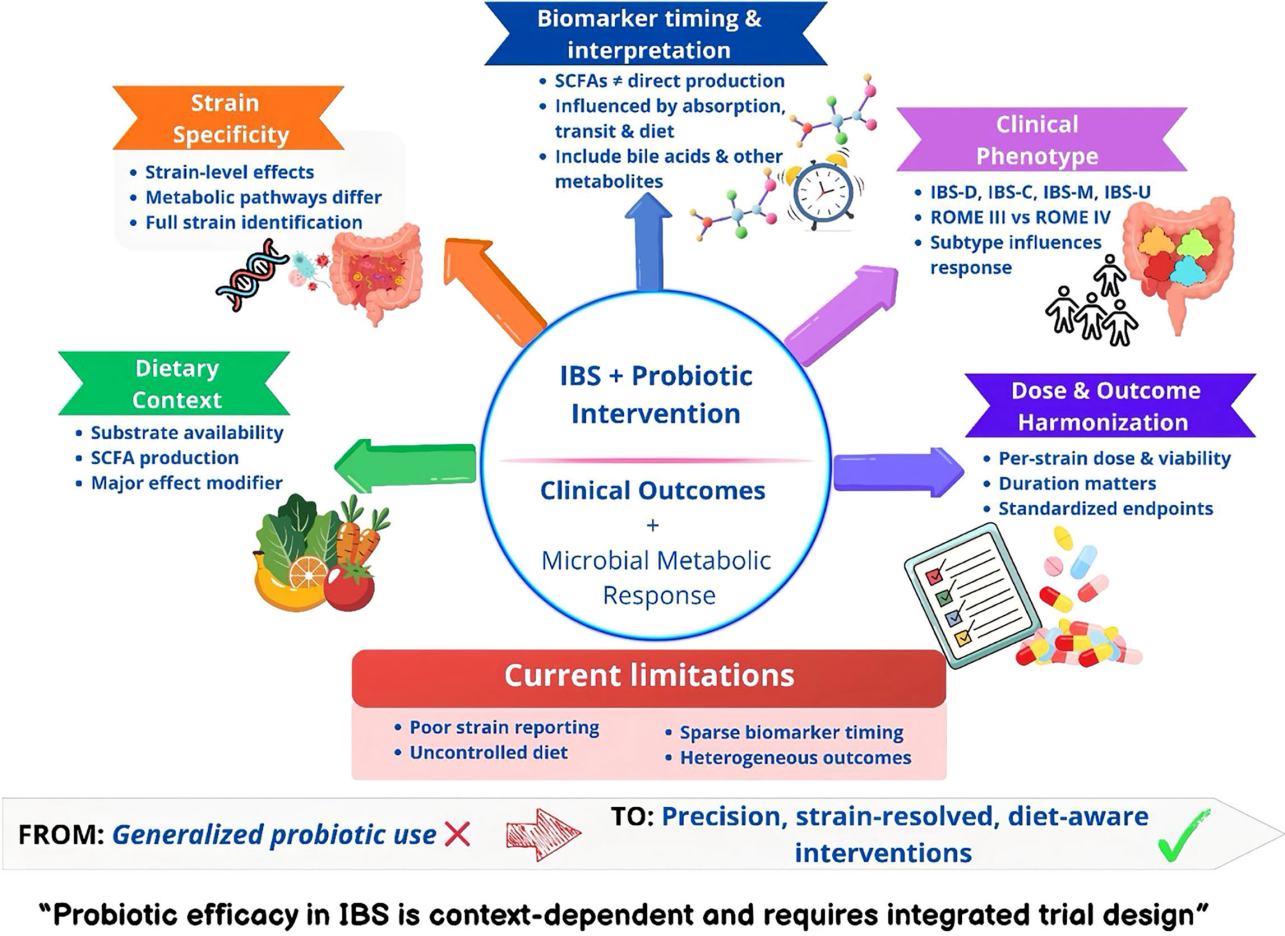
Conceptual framework for probiotic trial design and interpretation in irritable bowel syndrome (IBS). This graphical model illustrates how clinical outcomes and microbial metabolic responses arise from the interaction between IBS pathophysiology and probiotic exposure. Five key axes determine interpretability: (i) strain specificity, (ii) clinical phenotype and diagnostic criteria, (iii) dietary context, (iv) biomarker timing and metabolite interpretation, and (v) dose transparency and outcome harmonization. These domains interact to shape both biological response and clinical efficacy. The lower panel highlights common limitations in current probiotic IBS research, including incomplete strain characterization, inadequate dietary control, limited biomarker integration, and heterogeneous outcome definitions. Together, these factors underscore the need for precision, mechanism-informed, and standardized trial designs. This framework is operationalized as a practical checklist in **Table 2**.

To address these recurring methodological limitations, we propose a structured translational framework for probiotic trials in IBS. This framework integrates five key axes that determine interpretability and clinical relevance: a) strain identity and formulation specificity (Lacy et al., 2021; McFarland et al., 2021; Vasant et al., 2021; Goodoory et al., 2023; McFarland et al., 2023), b) definition of the clinical phenotype, including IBS subtype and diagnostic criteria (Gordon et al., 2003; Sheflin et al., 2017; Tanaka and Vécsei, 2025; Almalki et al., 2026), c) dietary context as a major modulator of microbial metabolism (Gordon et al., 2003; Vasant et al., 2021; Aggeletopoulou and Triantos, 2024; Clayson, 2025; Tanaka and Vécsei, 2025; Wang et al., 2025; Bogdanowska-Charkiewicz et al., 2026), d) mechanistic alignment through biomarker timeline and metabolite interpretation (Sheflin et al., 2017; Lacy et al., 2021; Vasant et al., 2021; McFarland et al., 2023; Clayson, 2025), and e) harmonization of results and dose transparency (Xie et al., 2023; Zampieri et al., 2023; Bang et al., 2025; Hoffmann Sardá et al., 2025; Zawistowska-Rojek et al., 2026). These axes interact to determine both the biological response and clinical outcomes and should therefore be considered integral components of trial design, rather than auxiliary descriptors. This framework is operationalized in **Table 2** as a practical checklist to improve the design, reporting, and interpretation of probiotic trials in IBS. Where feasible, future trials should incorporate functional genomics, pathway-resolved microbiome analysis, and metabolomic profiling to better connect microbial activity with host response. Equally important, future studies should explicitly report IBS subtype distribution and the diagnostic framework used, as these features are essential for cross-trial comparability and subtype-specific interpretation of probiotic effects. These interacting domains are summarized in a conceptual model (**Figure 1**), which illustrates how biological and methodological factors jointly determine the interpretability and clinical translation of probiotic interventions in IBS. It is important to note that this framework is conceptual and has not yet been prospectively validated in interventional studies. Rather than serving as a prescriptive model, it is intended to synthesize converging methodological insights from clinical trials, meta-analyses, and guideline-based recommendations. Its practical implementation will require prioritization based on study objectives, feasibility, and resource availability. Future prospective studies are needed to evaluate whether structured incorporation of these domains improves reproducibility, mechanistic interpretability, and clinical outcomes in probiotic trials.

**Table 2:** Translational trial design framework and checklist for probiotic studies in IBS.

Domain	Biological/methodological relevance	What should be reported or controlled	Minimum recommendation for future trials	Risk if omitted	References
Strain identity and formulation composition	Probiotic effects are strain-specific and formulation-dependent rather than class-wide	Full strain identifiers, formulation composition, manufacturer specification, and whether intervention is live probiotic, metabolite-derived, or postbiotic	Report complete strain-level identity for all components and clearly define formulation type	Replication becomes impossible, and findings cannot be meaningfully synthesized across studies	(Lacy et al., 2021; McFarland et al., 2021; Vasant et al., 2021; Goodoory et al., 2023; McFarland et al., 2023)
IBS subtype definition	IBS-D, IBS-C, IBS-M, and IBS-U differ in motility, symptom pattern, and potentially host–microbial interactions	Subtype distribution at baseline and subtype-specific analyses	Stratify randomization by IBS subtype or report prespecified subtype-stratified outcomes	True subtype-specific effects may be obscured by pooled analyses	(Gordon et al., 2003; Lacy et al., 2021; Vasant et al., 2021; Almalki et al., 2026)
Diagnostic criteria	Rome III and Rome IV define partially different patient populations and may influence observed heterogeneity	Diagnostic framework used, inclusion/exclusion criteria, and symptom severity thresholds	Explicitly report diagnostic criteria and maintain consistency across recruitment sites	Cross-trial comparability is reduced and pooled interpretation becomes less reliable	(Gordon et al., 2003; Lacy et al., 2021; Vasant et al., 2021)
Dose transparency	Nominal CFU values may not reflect delivered exposure, especially in multistrain products	Per-strain CFU, viability at administration, storage conditions, delivery matrix, and adherence-adjusted exposure	Report per-strain viable dose at point of use rather than only total dose at manufacture	Dose–response interpretation remains weak and reproducibility is compromised	(Zhang et al., 2022; McFarland et al., 2023; Bang et al., 2025; Zawistowska-Rojek et al., 2026)
Intervention duration	Clinical and biological responses may emerge at different time scales	Total intervention length, follow-up duration, and timing of outcome assessment	Use intervention periods sufficient to capture both early and sustained responses, ideally with post-intervention follow-up	Short trials may miss delayed benefit or misclassify transient effects	(Zhang et al., 2022; Bang et al., 2025)
Dietary context	Fermentable substrates, fiber type, and habitual diet strongly shape microbial metabolism and symptom expression	Dietary advice, dietary run-in, fiber/FODMAP exposure, and any dietary monitoring or standardization	Include standardized dietary frameworks, prospective intake assessment, or stratified analyses by dietary exposure	Diet-related changes may be misattributed to probiotics	(Sheflin et al., 2017; Aggeletopoulou and Triantos, 2024; Jent et al., 2024; Hoffmann Sardá et al., 2025; Bogdanowska-Charkiewicz et al., 2026; Rondinella et al., 2026)
Baseline microbiome and host context	Initial microbiota configuration and barrier/immune status may influence responsiveness	Baseline microbiome profiling, relevant host biomarkers, and clinical phenotype characterization	Incorporate baseline profiling to identify responder and non-responder phenotypes	Biological heterogeneity remains unexplained, and the mechanistic interpretation is weakened	(Aggeletopoulou and Triantos, 2024; Hoffmann Sardá et al., 2025; Jyoti, 2025; Li E. et al., 2025; Yang-Jensen et al., 2025)
Biomarker timing	SCFAs, inflammatory markers, and barrier indices are dynamic and may not align temporally with symptoms	Biomarker collection schedule relative to symptom assessment and intervention milestones	Synchronize serial biomarker sampling with clinical outcome time points	Mechanistic claims remain speculative due to poor temporal resolution	(Aggeletopoulou and Triantos, 2024; Clayson, 2025; Li X. et al., 2025; Wang et al., 2025; Yang-Jensen et al., 2025)
Metabolite interpretation	Fecal SCFAs are imperfect downstream readouts of microbial metabolism, as they are influenced by diet, epithelial absorption, intestinal transit, and luminal dilution, rather than reflecting net microbial production alone	Matrix used, timing of sample collection, dietary context, and interpretation limits; where possible include broader metabolite panels	Interpret SCFAs cautiously and complement them with additional metabolite classes such as bile acids and other functionally relevant microbial products	Overinterpretation of isolated fecal metabolite changes may generate misleading mechanistic conclusions	(Sheflin et al., 2017; Aggeletopoulou and Triantos, 2024; Jyoti, 2025; Liu et al., 2025; Rondinella et al., 2026)
Outcome harmonization	Heterogeneous symptom endpoints limit evidence synthesis and translational comparison	Primary outcome hierarchy, responder definitions, symptom tools, and assessment windows	Use core outcome sets, standardized symptom instruments, and prespecified clinically meaningful endpoints	Positive signals may appear inconsistent simply because studies measure different outcomes	(Gordon et al., 2003; McFarland et al., 2021; Zhang et al., 2022; Goodoory et al., 2023; Almalki et al., 2026)
Microbiome-focused mechanistic integration	Functional readouts may better explain response than taxonomy alone	Metabolomics, pathway-level microbiome analysis, functional genomics, and host–microbial biomarker integration where feasible	Incorporate multimodal mechanistic measures in a hypothesis-driven manner	Studies may remain clinically descriptive without clarifying microbial mechanisms of action	(Zampieri et al., 2023; Aggeletopoulou and Triantos, 2024; Jyoti, 2025; Kezer et al., 2025; Li X. et al., 2025; Tanaka and Vécsei, 2025)

Variability in probiotic efficacy across IBS studies likely reflects interacting biological and methodological modifiers rather than a simple absence of therapeutic effect. This framework is intended to improve reproducibility, interpretability, and translational value in future probiotic IBS trials. IBS, irritable bowel syndrome; IBS-D, diarrhea-predominant IBS; IBS-C, constipation-predominant IBS; IBS-M, mixed IBS; IBS-U, unsubtyped IBS; SCFA, short-chain fatty acid; CFU, colony-forming units; FODMAP, fermentable oligosaccharides, disaccharides, monosaccharides, and polyols.

## Biomarker timing and mechanistic interpretation

Although some probiotic IBS trials have incorporated serial clinical evaluations, biomarker assessment is often limited to a small number of time points, restricting mechanistic resolution across studies (Aggeletopoulou and Triantos, 2024; Li E. et al., 2025; Wang et al., 2025; Yang-Jensen et al., 2025). This design choice constrains mechanistic resolution. Processes central to IBS pathophysiology, including intestinal epithelial turnover, tight-junction remodeling, mucosal immune activation, and SCFA flux, are inherently dynamic and may evolve over days rather than weeks (Aggeletopoulou and Triantos, 2024; Yang-Jensen et al., 2025). Limited biomarker sampling therefore restricts the ability to establish temporal coupling between biological adaptation and symptomatic improvement, thereby weakening causal inference regarding the mechanism of action (Wang et al., 2025). This limitation is particularly relevant in IBS, where inflammation is typically low-grade or subclinical. Under such conditions, conventional inflammatory markers may lack sufficient sensitivity to capture modest yet biologically meaningful changes. The use of high-sensitivity inflammatory assays, combined with repeated measurement of epithelial junction proteins and fecal immune markers, would likely improve detection of probiotic-associated effects on mucosal homeostasis. Moreover, synchronizing biomarker acquisition with symptom assessment time points would allow more precise mapping of biological trajectories relative to clinical response (Clayson, 2025). Beyond static protein and cytokine measurements, longitudinal integration of metabolomic and microbiome-resolved analyses is essential to distinguish sustained metabolic reprogramming from transient adaptive responses. However, even temporally resolved biomarker strategies remain insufficient if the primary drivers of microbial metabolism are not concurrently controlled. In IBS, biological signals attributed to probiotic intervention are continuously shaped by dietary substrates that determine fermentation dynamics, SCFA availability, and epithelial exposure. Fecal SCFA concentrations should not be interpreted as direct proxies of net microbial production. They reflect not only fermentation activity, but also epithelial absorption, intestinal transit time, luminal dilution, and recent dietary exposure (den Besten et al., 2013; Sakata, 2019; LaBouyer et al., 2022). Moreover, microbiome-related signaling in IBS is unlikely to be captured by SCFAs alone, as bile acid derivatives and other microbially modified metabolites may also contribute to motility, epithelial permeability, immune tone, and symptom generation (Xiao et al., 2021; Min et al., 2022; Shute et al., 2022; Li X. et al., 2025). As a result, limitations in biomarker timing intersect with dietary variability, generating compounded interpretive uncertainty and positioning diet not as a secondary covariate, but as a major effect modifier that must be explicitly addressed to contextualize probiotic efficacy. A microbiome-focused interpretation should not rely exclusively on symptom trajectories or isolated fecal metabolite measurements. Functional characterization of the microbial ecosystem is also needed to clarify whether observed responses reflect altered substrate utilization, cross-feeding interactions, or broader pathway-level reprogramming. In this context, longitudinal integration of targeted or untargeted metabolomics with metagenomic, metatranscriptomic, or other functional profiling approaches would provide a more mechanistically informative framework for interpreting probiotic effects in IBS (Jacobs et al., 2023; Puig-Castellví et al., 2023; Shin and Kashyap, 2023; Butowski et al., 2025). Such strategies may help distinguish taxonomic change from functional adaptation and identify whether shifts in microbial metabolism are transient, compensatory, or linked to clinically meaningful response.

## Dietary context as a major effect modifier

Dietary intake represents one of the most powerful and immediate modulators of IBS symptom expression and gut microbial metabolic output, yet it remains inconsistently characterized and insufficiently controlled across probiotic intervention trials (Aggeletopoulou and Triantos, 2024; Li X. et al., 2025). Fermentable carbohydrates, fiber composition, and overall dietary patterns exert direct and potent effects on luminal SCFA production, osmotic balance, epithelial permeability, and mucosal immune signaling, which are the same biological pathways commonly ascribed to probiotic mechanisms of action (Rondinella et al., 2026). However, failure to rigorously define dietary exposure fundamentally limits causal attribution. Clinical and network meta-analytic evidence consistently demonstrates that structured dietary interventions, particularly low-fermentable oligosaccharides, disaccharides, monosaccharides, and polyols (FODMAP, LFD) approaches, produce clinically meaningful improvements in abdominal pain, bloating, and global symptom severity in IBS (Aggeletopoulou and Triantos, 2024; Jent et al., 2024; Kuźmin et al., 2025; Bogdanowska-Charkiewicz et al., 2026). Moreover, dietary modulation on its own can drive pronounced alterations in gut microbial composition and metabolic output, including shifts in key SCFA such as acetate, propionate, and butyrate (Sheflin et al., 2017; Jyoti, 2025). Against this background, reliance on generalized dietary advice and retrospective dietary recording is insufficient to exclude diet-driven contributions to observed symptom improvement and biomarker change. Beyond its role as a clinical confounder, dietary context is also a major determinant of microbial functional output. Fermentable substrates influence pathway activation, metabolite exchange among microbial taxa, and the balance between saccharolytic and other metabolic processes (Verbeke et al., 2015; Korpela, 2018; Rowland et al., 2018; Jardon et al., 2022; Culp and Goodman, 2023). Accordingly, probiotic efficacy in IBS may depend not only on the administered strain itself, but also on the surrounding metabolic network that determines substrate availability, ecological compatibility, and downstream metabolite production.

Without standardized dietary run-in periods, quantitative assessment of fermentable substrate intake, or stratification by fiber type and load, probiotic-associated effects cannot be disentangled from concurrent diet-induced microbial and metabolic adaptations (Tanaka and Vécsei, 2025). This limitation is particularly relevant when changes in SCFA concentrations are interpreted as mechanistic evidence of probiotic efficacy, despite the well-established sensitivity of these metabolites to short-term dietary variation.

Future probiotic trials in IBS must therefore move beyond minimal dietary guidance and adopt structured dietary frameworks as integral components of study design. Approaches incorporating dietary standardization, stratified randomization based on fermentable carbohydrate intake, or factorial designs combining diet and probiotic interventions would substantially enhance internal validity (Hoffmann Sardá et al., 2025). Such designs would not only reduce systematic confounding, but also enable identification of responder phenotypes in which probiotic efficacy is conditional upon dietary context. Addressing diet as a major effect modifier is thus essential for advancing probiotic research in IBS from associative outcomes toward reproducible, mechanism-informed, and clinically actionable interventions.

## Strain-specific effects and translational limitations

Both the British Society of Gastroenterology (Vasant et al., 2021) and the American College of Gastroenterology (Lacy et al., 2021) emphasize that evidence supporting probiotics in IBS is inconsistent and should not be generalized across products. This position is strongly supported by strain-resolved systematic reviews and network meta-analyses, which consistently demonstrate that efficacy varies at the strain level, not merely by genus or species (McFarland et al., 2023; Al-Aklabi et al., 2026). Meta-analytic and mechanistic evidence indicate that only a small subset of microorganisms, specifically *Lactobacillus plantarum* 299v*, Bacillus coagulans* MTCC 5260, and *Saccharomyces boulardii* CNCM I 745, demonstrate reproducible and outcome-specific benefits in IBS, although these effects remain modest and highly context dependent (Gu et al., 2022; Xie et al., 2023; Lim and Lim, 2025). However, reporting probiotic interventions only at the genus level, or without full strain and formulation-level characterization, constitutes a significant translational limitation across probiotic research because it prevents replication, limits evidence synthesis, and restricts incorporation into clinical guideline development (Gordon et al., 2003; McFarland et al., 2021; Goodoory et al., 2023; Hoffmann Sardá et al., 2025; Tanaka and Vécsei, 2025; Maslennikov et al., 2026). Precision microbiome therapeutics in IBS requires equally precise reporting. Strain specificity is also relevant at the functional level. Closely related strains may differ in substrate utilization, metabolite production capacity, epithelial signaling effects, and interactions with resident microbial communities (Thomas and Versalovic, 2010; Douillard et al., 2013; Li B. et al., 2025). These differences may translate into distinct effects on host pathways related to epithelial permeability (Ferris et al., 2025), mucosal immune tone, and gut–brain signaling, including tight-junction remodeling, NF-κB and MAPK inflammatory cascades (Thomas and Versalovic, 2010), SCFA-linked signaling, bile acid–responsive pathways, and serotonergic regulation of motility and visceral hypersensitivity (Gu et al., 2022; Li X. et al., 2025; Tang et al., 2026). For this reason, strain characterization at the level of metabolic pathways and metabolites is increasingly necessary to understand why seemingly similar probiotic interventions produce different clinical outcomes (Thomas and Versalovic, 2010; Lee et al., 2025). Beyond strain identity, probiotic response in IBS is strongly modulated by dietary context, baseline microbiota composition, barrier integrity, and outcome timing, factors that collectively explain much of the heterogeneity observed across clinical trials (**Table 2**).

## Dose transparency and outcome harmonization

Quantitative transparency remains a central methodological weakness in probiotic research and a major contributor to heterogeneity in IBS trial outcomes (Bang et al., 2025). Reporting practices that provide only aggregate colony-forming unit (CFU) values at manufacture, or minimum CFU per dose without specifying per-strain viability, stability across shelf life, and viable counts at the point of ingestion, substantially limit assessment of dose–response relationships and impair reproducibility across studies (Zawistowska-Rojek et al., 2026). In meta-regression analyses of IBS probiotic trials, treatment duration, but not nominal dose, has been identified as a significant modifier for selected symptom outcomes, underscoring the need for both precise exposure reporting and duration standardization when interpreting efficacy (Zhang et al., 2022).

These limitations are particularly pronounced in multistrain or metabolite-producing formulations, where biological activity may depend less on nominal CFU counts and more on strain-specific growth dynamics, metabolite production capacity, and survival through gastrointestinal transit (Zampieri et al., 2023). To support mechanistic inference and enable meaningful meta-analytic modeling, future trials should report, at minimum, full strain identifiers, per-strain CFU targets, independent verification of viable counts at baseline and end of storage, delivery matrix and storage conditions, and adherence-adjusted estimates of received dose.

Outcome heterogeneity further constrains evidence synthesis and clinical translation. IBS trials variably prioritize global symptom relief, severity indices, abdominal pain, bloating, stool characteristics, and quality-of-life measures, often without prespecified outcome hierarchies or harmonized assessment windows (Gordon et al., 2003). This variability complicates cross-study comparison and can obscure subtype-specific effects, particularly given established biological and clinical distinctions among diarrhea-predominant, constipation-predominant, mixed, and unsubtyped (Gordon et al., 2003). This problem is further amplified when trials enroll mixed IBS populations without consistent subtype stratification or apply different diagnostic frameworks, as Rome III and Rome IV do not necessarily capture identical patient groups. Consequently, pooled estimates may reflect differences in case definition and subtype composition as much as differences in probiotic efficacy. Adoption of core outcome sets with standardized instruments, prespecified responder definitions, and subtype-stratified analyses would improve interpretability. Aligning clinical endpoints temporally with biomarker acquisition would further strengthen linkage between mechanistic changes, such as SCFA dynamics, epithelial permeability, and immune signaling, and clinically meaningful response, thereby advancing translation toward precision-guided probiotic therapy in IBS.

## Discussion

Recent probiotic studies in IBS, including trials incorporating metabolite-focused approaches, provide valuable insight into the role of microbial metabolism in symptom modulation and barrier function. However, when considered alongside strain-resolved meta-analyses, systematic reviews, and clinical guidelines, the broader literature also reveals persistent structural limitations that constrain translational applicability, including incomplete strain characterization, insufficient dietary control, sparse biomarker timing, and heterogeneous outcome definitions (Gordon et al., 2003; Sheflin et al., 2017; McFarland et al., 2021; Goodoory et al., 2023; Hoffmann Sardá et al., 2025; Lim and Lim, 2025; Almalki et al., 2026; Maslennikov et al., 2026).

First, probiotic IBS trials frequently exhibit a mismatch between mechanistic ambition and methodological control. Improvements in fecal SCFA levels and epithelial markers are often interpreted as evidence of probiotic efficacy. However, as discussed above, fecal SCFA measurements are influenced by multiple physiological and dietary factors and should not be interpreted as direct proxies of microbial production. Accordingly, mechanistic inference based solely on SCFA changes remains limited in the absence of controlled dietary exposure and temporally resolved biomarker assessment (Sheflin et al., 2017; Min et al., 2022; Jacobs et al., 2023; Aggeletopoulou and Triantos, 2024; Li X. et al., 2025). In addition, a metabolite-centered interpretation of IBS should extend beyond SCFAs alone, as microbiota-related effects may involve bile acid metabolism and other small-molecule mediators relevant to motility, barrier integrity, immune activation, and visceral signaling. Given the strong and well-established influence of diet on both symptom expression and microbial metabolism, failure to control or stratify dietary exposure remains a major limitation in attributing observed effects to probiotic interventions (Aggeletopoulou and Triantos, 2024; Lim and Lim, 2025; Al-Aklabi et al., 2026). In the absence of controlled dietary exposure and temporally dense biomarker sampling, mechanistic interpretation remains vulnerable to confounding. As a result, it remains unclear whether observed biological changes represent direct effects of the intervention, secondary consequences of altered diet–microbiota dynamics, or transient adaptive responses rather than sustained reprogramming.

A second major limitation across probiotic IBS research is incomplete biological characterization of the intervention. Consistent with prior sections, the available evidence supports a strain-specific rather than class-wide interpretation of probiotic efficacy, underscoring the need for precise intervention characterization. Failure to report full strain identity, per-strain dose, and viability at ingestion prevents replication, impairs quantitative synthesis, and ultimately precludes incorporation into guideline-based care. It is important to note that incomplete information at the strain level does not inherently invalidate biologically plausible mechanistic hypotheses; rather, it limits their reproducibility and integration into precision-guided clinical frameworks. Precision microbiome interventions require accurate information and clearly defined intervention parameters. The above does not imply that metabolite- or postbiotic-based strategies are inherently inferior to interventions using live strains, but rather that insufficient biological characterization compromises reproducibility and hinders the proper integration of clinical guidelines.

Third, outcome heterogeneity and limited alignment between clinical endpoints and mechanistic readouts continue to dilute interpretability across studies. Without harmonized outcome hierarchies, subtype-aware analyses, and temporal synchronization between symptom trajectories and biological markers, causal pathways remain speculative. This limitation is particularly consequential in IBS, where heterogeneity across diarrhea-predominant, constipation-predominant, mixed, and unsubtyped phenotypes is well established and likely reflects distinct pathophysiological substrates.

In addition, interpretability is further reduced when diagnostic criteria are inconsistently applied across trials, because Rome-based case definitions may shape baseline symptom profiles, subtype distribution, and the apparent magnitude of response (Gordon et al., 2003; Vasant et al., 2021; Almalki et al., 2026). Beyond these considerations, current probiotic IBS research remains limited by a mechanistic interpretation that is often insufficiently microbiome-centered. Clinical response is still discussed more often in relation to symptoms and broad inflammatory or barrier markers than to microbial functional activity, even though probiotic effects are likely mediated through pathway-level interactions involving substrate utilization, cross-feeding, metabolite exchange, and host–microbial signaling. Greater integration of metabolomics, functional genomics, and pathway-resolved microbiome analyses would strengthen mechanistic inference and better align probiotic trial design with the biological complexity of IBS (Jacobs et al., 2023; Shin and Kashyap, 2023; Sherwani et al., 2025). Such approaches may also help identify responder phenotypes based on microbial function rather than taxonomy alone.

Collectively, these considerations underscore a broader conceptual limitation in how probiotic interventions are currently investigated and interpreted in IBS. Probiotic research has often remained additive rather than integrative, incorporating biological measurements into conventional trial designs without restructuring those designs around the main determinants of microbial function. Diet, strain identity, dose exposure, biomarker timing, and clinical phenotype are not ancillary variables but key determinants of interpretability and response. When they are inadequately specified or controlled, even well-executed trials may generate signals that are biologically interesting yet clinically ambiguous. Progress will therefore require a shift away from generalized probiotic efficacy claims toward hypothesis-driven, strain-resolved interventions embedded within controlled dietary frameworks and evaluated with mechanism-aligned, subtype-aware outcomes. Importantly, the considerations discussed here are not derived from a single trial, but from converging evidence across randomized studies, strain-resolved meta-analyses, and clinical guidelines in IBS. Under such a framework, microbiome-based interventions may be interpreted with greater precision and translated into more clinically meaningful strategies.
